# Risdiplam utilization, adherence, and associated health care costs for patients with spinal muscular atrophy: a United States retrospective claims database analysis

**DOI:** 10.1186/s13023-024-03399-0

**Published:** 2024-12-30

**Authors:** Anish Patel, Walter Toro, Min Yang, Wei Song, Raj Desai, Mingchen Ye, Nadia Tabatabaeepour, Omar Dabbous

**Affiliations:** 1 Novartis Gene Therapies, Inc., 2275 Half Day Road, Suite 200, Bannockburn, IL 60015 USA; 2 Analysis Group, Inc., 111 Huntington Ave, Floor 13, Boston, MA 02199 USA; 3 Analysis Group, Inc., 650 California Street, Floor 23, San Francisco, CA 94108 USA

**Keywords:** Adherence, Persistence, Claims database analysis, Health care costs, Health care utilization, Risdiplam, Spinal muscular atrophy, United States

## Abstract

**Background:**

Spinal muscular atrophy (SMA) is a genetic neuromuscular disease associated with progressive loss of motor function. Risdiplam, a daily oral therapy, was approved in the United States for the treatment of SMA. Risdiplam’s effectiveness depends on patient adherence to the treatment regimen. This retrospective claims database analysis assessed real-world treatment adherence and persistence, and all-cause health care costs by adherence status, for patients with SMA receiving risdiplam. Outcomes were summarized by SMA types (1−4) and age groups (0–2, 3–5, 6–17, and ≥ 18 years).

**Results:**

86 patients with ≥ 1 SMA diagnosis, risdiplam treatment, and ≥ 6 months of continuous enrollment after the index date (SMA diagnosis) were identified in the IQVIA PharMetrics^®^ Plus database (01/01/2020−06/30/2022). One patient had SMA type 1 (a 1-year-old boy), 18 had type 2 (mean ± SD age: 7.9 ± 5.7 years; 61% female), 47 had type 3 (17.3 ± 10.2 years; 55% female), and 20 had type 4 (38.2 ± 11.6 years; 55% female). The mean proportion of days covered (PDC) with risdiplam was 0.89 overall, ranging from 0.88 for SMA type 4 to 0.97 for type 1. The majority (83.7%) of patients were adherent to risdiplam (PDC ≥0.80), ranging from 75.0% for type 4 to 100% for type 1. Adherence ranged from 76.5% among 6–12-year-olds to 100% among 0–2-year-olds. Compared with adherent patients, nonadherent patients had higher median total health care costs by $335,049 for type 2, $41,204 for type 3, and $12,223 for type 4. Among adherent patients, patients with PDC between 0.90 and 1.00 had lower costs compared with patients with PDC between 0.80 and 0.90.

**Conclusions:**

Nonadherence to risdiplam was observed in the first year of treatment, especially for patients with SMA type 4 and patients aged 6–12 years. Nonadherence was associated with higher all-cause health care costs, with the most pronounced cost difference for SMA type 2. For adherent patients, those who were highly adherent incurred lower health care costs. These findings underscore the importance of treatment adherence and persistence for patients with SMA receiving risdiplam, particularly for younger children and those with greater disease severity.

**Supplementary Information:**

The online version contains supplementary material available at 10.1186/s13023-024-03399-0.

## Background

Spinal muscular atrophy (SMA) is a rare but severe neurogenetic disorder primarily caused by loss of or mutation in the *survival motor neuron 1* (*SMN1*) gene on chromosome 5q13, which leads to degeneration of α-motor neurons with resulting muscular atrophy and impairment of motor neuron function [1]. SMA is typically diagnosed in early childhood, with an estimated prevalence at birth of 8.5–10.3 per 100,000 live births, and prior to the approval of the disease-modifying treatments (DMTs), it was the most common genetic cause of infant death [2–5]. SMA encompasses a wide range of clinical severity and has been classified into subtypes according to the age at disease onset, motor function achieved, and prognosis, ranging from the most severe (SMA type 0) to the mildest (SMA type 4) form [6, 7]. SMA type 0 (prenatal onset) and SMA type 1 (infantile onset) are the most common and severe forms, SMA types 2 and 3 have a later onset and a less severe course, and SMA type 4 (adult onset) is the rarest and least severe [8, 9]. Patients with SMA typically experience a rapid decline in motor function soon after symptom onset followed by plateau and stabilization periods, although the decline in function is irreversible [5, 10].

Three DMTs have been approved by the United States Food and Drug Administration (FDA) for the treatment of SMA: nusinersen [11], onasemnogene abeparvovec [12], and risdiplam [13]. All three DMTs increase full-length SMN protein levels (i.e., the protein produced by *SMN1*) via distinct mechanisms and use different routes of administration. Nusinersen, an intrathecal therapy, was approved by the FDA in December 2016 to treat pediatric and adult patients with SMA [11]. After four loading doses, nusinersen requires a maintenance dose once every 4 months. Onasemnogene abeparvovec, a one-time single-dose intravenous infusion, was approved by the FDA in May 2019 for the treatment of pediatric patients aged ≤2 years with SMA and biallelic mutations in *SMN1* [12]. In August 2020, risdiplam, a daily oral medication, was approved by the FDA to treat SMA in patients aged ≥2 months [13].

For chronic diseases, medication adherence (the degree to which the dosing schedule is followed) [14] and persistence (continuing use of the prescribed therapy) [15] have been shown to contribute to long-term treatment effectiveness in real-world practice. As ongoing therapies, both nusinersen and risdiplam presumably require lifelong use to confer the maximal benefit. However, several real-world studies of nusinersen adherence and persistence for patients with SMA have found that the adherence to nusinersen is low and could potentially lead to negative clinical and economic outcomes, including greater frequency of comorbidities, a higher rate of hospitalization, and increased medical costs [16–19].

Given the recent approval of risdiplam, there is limited real-world evidence of the adherence or persistence with risdiplam for patients with SMA, or the impact of nonadherence on patients’ health care costs. A clear understanding of the current treatment patterns and health care costs for patients with SMA treated with risdiplam may improve the clinical management of this disabling disease and inform treatment decision-making by health care providers and other stakeholders. To address this gap in the literature, the objectives of the current study were to (1) describe the treatment patterns for patients with SMA receiving risdiplam, including their treatment regimen, adherence, discontinuation, and persistence, and (2) to describe the all-cause health care costs in patients who were adherent and nonadherent to risdiplam. Further, these analyses were stratified by SMA type to inform the potential association related to SMA severity. In addition, treatment adherence was examined across age groups (0–2 years, 3–5 years, 6–12 years, 13–17 years and ≥ 18 years).

## Methods

### Data source

This study used a large US health insurance claims database (IQVIA PharMetrics^®^ Plus) and included data spanning from January 1, 2020, to June 30, 2022. The database is comprised of fully adjudicated medical and pharmacy claims for over 210 million unique enrollees since 2006. It is representative of the commercially insured US national population for patients under 65 years of age and contains a longitudinal view of inpatient and outpatient services, prescription and office/outpatient administered drugs, provider specialty, costs including allowed and paid amounts, patient demographics (including state), and detailed enrollment information.

This study did not include any access to identifiable personal information and thus did not require institutional review board review and approval.

### Study population

**Fig. 1:**
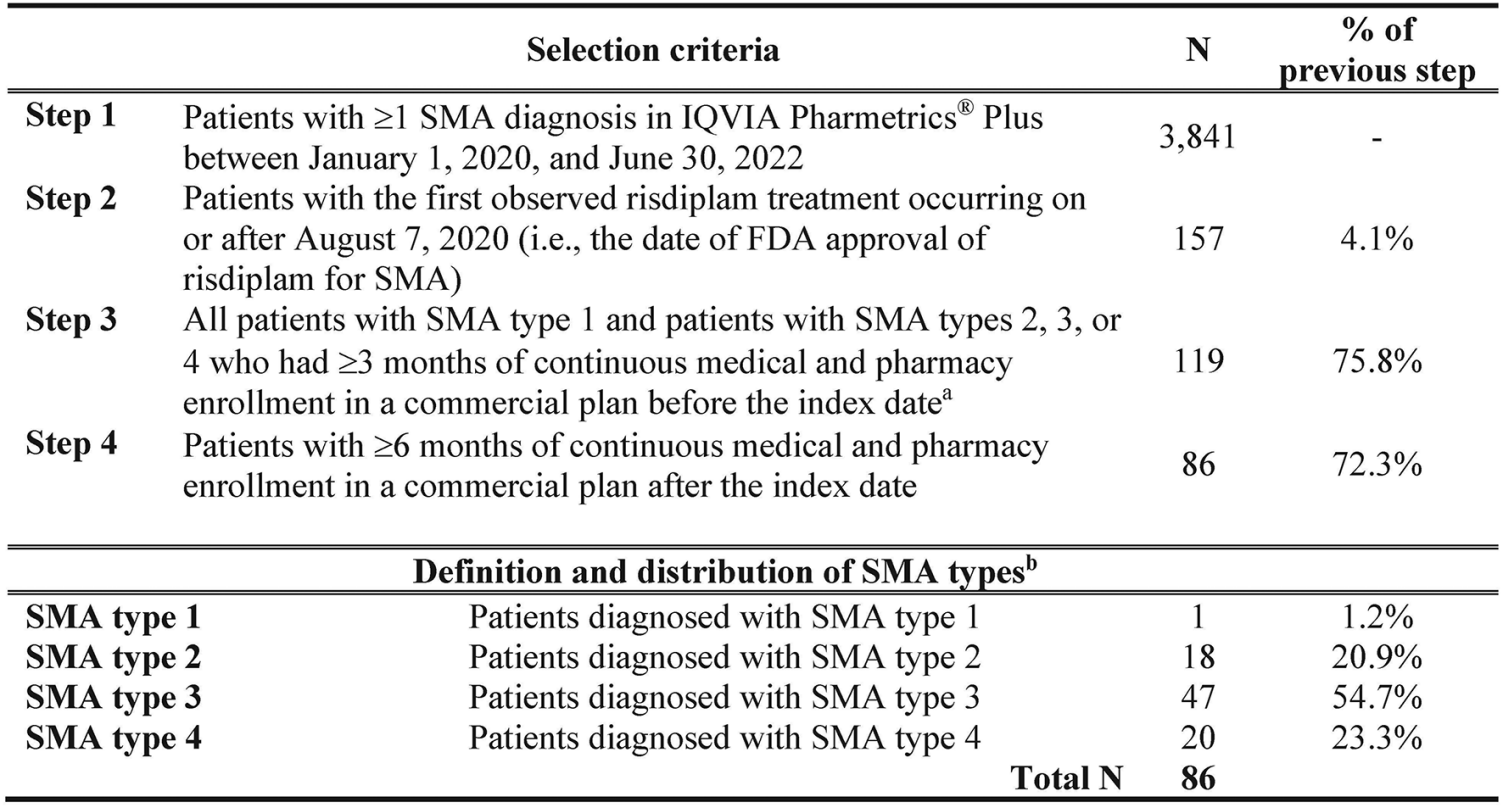
Sample selection of patients with SMA who received risdiplam. FDA, Food and Drug Administration; SMA: spinal muscular atrophy. ^a^The index date was defined as initiation of risdiplam. ^b^Patients with SMA were classified into four SMA types using the algorithm presented in Tables S1 and S2

The study selection criteria are described in Fig. 1. Patients who met all the following criteria were eligible for inclusion: (1) ≥ 1 SMA diagnosis (International Classification of Disease, Tenth Revision, Clinical Modification [ICD-10-CM] diagnosis codes: G12.0, G12.1, G12.9) between January 1, 2020, and June 30, 2022; (2) treated with risdiplam on or after August 7, 2020 (i.e., the date of risdiplam approval by the FDA); and (3) ≥ 6 months of continuous enrollment after the index date, defined as the date of risdiplam initiation. In addition, patients with SMA types 2, 3, or 4 were required to have ≥ 3, months of continuous enrollment before the index date.

The study period was defined as the time from the index date to the end of continuous enrollment, the end of data availability (i.e., June 30, 2022), or the end of 1-year follow-up, whichever occurred first. Fig. S1 provides an overview diagram of the study design.

### Identification of SMA types

SMA types were identified using an established claims-based algorithm based on age at first SMA diagnosis, age recorded at the end of continuous clinical activity or end of data availability, and symptoms, procedures, and/or the use of durable medical equipment (DME) [20–22]. The details of the algorithm are presented in Table S1, and the drug and procedure codes are listed in Table S2.

### Patient characteristics

Patient characteristics were summarized separately for patients with SMA by type. Demographics were summarized as of the index date and included the following variables: age at index, sex, and US Census region (South, Midwest, West, Northeast). Clinical characteristics were summarized during the study period and included SMA-related comorbidities (e.g., scoliosis, dyspnea and respiratory anomalies, muscle weakness [generalized], chronic respiratory failure, feeding difficulties and mismanagement) and other comorbidities (e.g., neurodevelopmental disorders, elimination disorders, anxiety disorders, depressive disorders, other conditions warranting clinical attention).

### Study outcomes

Study outcomes included treatment patterns (i.e., treatment regimens, adherence to risdiplam, risdiplam discontinuation and persistence) and all-cause health care costs during the study period.

#### Treatment patterns

##### Risdiplam treatment regimen

Treatment regimens of risdiplam with/without nusinersen or onasemnogene abeparvovec were described using all available data from January 1, 2020, to June 30, 2022, and identified with the codes listed in Table S3.

##### Adherence to risdiplam

The proportion of days covered (PDC) was used to measure adherence to risdiplam in the study period. PDC was calculated as the total number of days covered with risdiplam during the study period divided by the number of days in the study period. The number of days of supply from the last risdiplam prescription beyond the end of the study period was truncated. If a patient received a subsequent risdiplam dispensing earlier than expected, the number of days overlapping with the previous risdiplam days of supply was counted as one day each “with drug on hand” in the PDC calculation. PDC ranged from 0 to 1 and was dichotomized and used to classify patients as adherent (PDC ≥ 0.80) or nonadherent (PDC < 0.80) [23]. Adherence by age group (i.e., age 0–2 years, 3–5 years, 6–17 years, ≥ 18 years) was also described.

##### Risdiplam discontinuation and persistence

Discontinuation was defined as no claim for risdiplam within 30 days after the previous days of supply was exhausted, switching to nusinersen, or receiving treatment with onasemnogene abeparvovec. The date of discontinuation was set as the last days of supply of risdiplam before reaching the 30-day allowable gap or the date of nusinersen initiation or treatment with onasemnogene abeparvovec, whichever came first. Persistence was defined as the time from the index date to the date of risdiplam discontinuation. Patients remaining on treatment at the end of study period were censored.

#### All-cause health care costs

Given the small sample sizes and the wide range of health care services that patients with SMA may receive, all-cause total health care costs (including medical services and pharmacy costs) were reported and described for both adherent and nonadherent patient populations. Medical services costs included all costs associated with inpatient, outpatient, emergency department (ED), and other medical visits (i.e., DME, dental and vision care, home health). Costs associated with mechanical ventilation support and nutritional support were also reported as part of the medical services costs. Pharmacy costs included all costs associated with any prescription medications. SMA treatment costs (costs of nusinersen and onasemnogene abeparvovec recorded in medical claims and costs of risdiplam recorded in pharmacy claims) were excluded from the total medical services or pharmacy costs.

Health care costs were summarized on a per-patient per-year (PPPY) basis, which were calculated as the costs divided by patient-years of follow-up in the study period for each patient. Costs were inflated to 2023 US dollars (USD) using the Personal Consumption Expenditures Health Index from the US Bureau of Economic Analysis [24].

### Statistical analyses

Descriptive statistics were used to characterize risdiplam treatment regimens for all eligible patients and adherence to risdiplam by SMA type. Continuous variables were described using means, standard deviations (SDs), and medians (interquartile range [IQR]); categorical variables were summarized using counts and proportions. Treatment persistence with risdiplam was analyzed using the Kaplan Meier method. All-cause health care costs were described for the risdiplam-adherent and nonadherent patient populations by SMA type, and assessed based on PDC levels (< 0.80, 0.80–<0.90, and 0.90–1.00). No statistical comparisons between cohorts were conducted. All analyses were conducted in RStudio, version 4.2.2.

## Results

### Sample selection and patient characteristics

A total of 86 patients met all criteria and were included in the study. Of these patients, one had SMA type 1 (a 1-year-old boy), 18 had type 2 (mean ± SD age: 7.9 ± 5.7 years; 61% female), 47 had type 3 (17.3 ± 10.2 years; 55% female), and 20 had type 4 (38.2 ± 11.6 years; 55% female) (Fig. 1; Table 1). SMA-related comorbidities were common in the study cohort and for those with SMA types 2 and 3; more than 70% had scoliosis and approximately 45% had dyspnea and respiratory anomalies. Generalized muscle weakness was observed in 66.7% of SMA type 2 patients, 25.5% of SMA type 3, and 25.0% of SMA type 4 patients.

**Table 1:** Demographics and clinical characteristics of patients with SMA types 1–4

Patient characteristics	SMA type 1 (*n* = 1)	SMA type 2 (*n* = 18)	SMA type 3 (*n* = 47)	SMA type 4 (*n* = 20)
**Demographic characteristics** ^a^				
Age at index date,^b^ years				
Mean ± SD	1.0 ± NA	7.9 ± 5.7	17.3 ± 10.2	38.2 ± 11.6
Median (IQR)	1.0 (1.0, 1.0)	5.5 (4.0, 10.0)	17.0 (10.0, 21.0)	37.0 (28.0, 48.0)
Sex, n (%)				
Female	0 (0.0%)	11 (61.1%)	26 (55.3%)	11 (55.0%)
US Census region, n (%)				
South	1 (100.0%)	4 (22.2%)	17 (36.2%)	13 (65.0%)
Midwest	0 (0.0%)	11 (61.1%)	13 (27.7%)	3 (15.0%)
West	0 (0.0%)	2 (11.1%)	10 (21.3%)	1 (5.0%)
Northeast	0 (0.0%)	1 (5.6%)	7 (14.9%)	3 (15.0%)
**Clinical characteristics** ^c^				
SMA-related comorbidities, n (%)	1 (100.0%)	16 (88.9%)	42 (89.4%)	12 (60.0%)
Scoliosis	1 (100.0%)	13 (72.2%)	33 (70.2%)	0 (0.0%)
Dyspnea and respiratory anomalies	1 (100.0%)	8 (44.4%)	21 (44.7%)	1 (5.0%)
Muscle weakness (generalized)	0 (0.0%)	12 (66.7%)	12 (25.5%)	5 (25.0%)
Chronic respiratory failure	1 (100.0%)	8 (44.4%)	15 (31.9%)	1 (5.0%)
Feeding difficulties and mismanagement	0 (0.0%)	8 (44.4%)	9 (19.1%)	1 (5.0%)
Other comorbidities, n (%)	1 (100.0%)	9 (50.0%)	17 (36.2%)	9 (45.0%)
Neurodevelopmental disorders	1 (100.0%)	7 (38.9%)	7 (14.9%)	1 (5.0%)
Elimination disorders^d^	0 (0.0%)	2 (11.1%)	7 (14.9%)	1 (5.0%)
Anxiety disorders	0 (0.0%)	0 (0.0%)	4 (8.5%)	5 (25.0%)
Depressive disorders	0 (0.0%)	0 (0.0%)	3 (6.4%)	5 (25.0%)
Other conditions that may be a focus of clinical attention	0 (0.0%)	2 (11.1%)	2 (4.3%)	3 (15.0%)

IQR, interquartile range; NA, not applicable; SD, standard deviation; SMA, spinal muscular atrophy; US, United States

^a^Demographic characteristics were summarized as of the index date

^b^The index date was defined as the date of risdiplam initiation on or after August 7, 2020 (i.e., the date when risdiplam was approved for the treatment of SMA in pediatric and adult patients in the United States)

^c^Clinical characteristics were summarized during the study period defined as the time from the index date to the end of continuous enrollment, the end of data availability (i.e., June 30, 2022), or the end of 1-year follow-up, whichever occurred first

^d^Elimination disorders involve the inappropriate elimination of urine or feces and are first diagnosed in childhood or adolescence

### Treatment patterns

#### Risdiplam treatment regimens

A total of 43 patients (50.0%) were treated with risdiplam monotherapy while 42 (48.8%) received nusinersen prior to risdiplam; nine of these patients switched back to nusinersen. One patient received treatment with risdiplam after nusinersen and onasemnogene abeparvovec.

#### Adherence to risdiplam dosing schedule

The mean PDC was 0.89 for the overall cohort, ranging from 0.88 for patients with SMA type 4 to 0.97 for the patient with SMA type 1 (Table 2). The majority (83.7%) of patients were adherent to risdiplam (PDC ≥ 0.80) although the adherence percentage varied across different SMA types, ranging from 75.0% for SMA type 4 to 100% for SMA type 1. The level of adherence also varied by age group and was greatest for those aged 0–2 years (mean PDC: 0.96) and lowest for patients aged 6–12 years (mean PDC: 0.85). Similarly, the percentage of patients who were adherent to risdiplam ranged from 76.5% for those aged 6–12 years to 100% for those aged 0–2 years.

**Table 2:** Adherence to risdiplam among patients with SMA overall, by SMA type, and by age groups

Adherence	Overall (*N* = 86)	SMA type				Age group^a^				
		Type 1 (*n* = 1)	Type 2 (*n* = 18)	Type 3 (*n* = 47)	Type 4 (*n* = 20)	0–2 years (*n* = 3)	3–5 years (*n* = 10)	6–12 years (*n* = 17)	13–17 years (*n* = 15)	≥ 18 years (*n* = 41)
PDC,^b^ mean ± SD	0.89 ± 0.18	0.97 ± NA	0.89 ± 0.18	0.90 ± 0.19	0.88 ± 0.19	0.96 ± 0.04	0.92 ± 0.15	0.85 ± 0.24	0.88 ± 0.23	0.91 ± 0.16
PDC,^b^ median (IQR)	0.98 (0.89, 1.00)	0.97 (NA)	0.98 (0.91, 1.00)	0.98 (0.88, 1.00)	0.96 (0.86, 0.99)	0.97 (0.91, 1.00)	0.98 (0.91, 0.99)	0.97 (0.86, 1.00)	0.97 (0.88, 1.00)	0.98 (0.91, 0.99)
Adherent patients (PDC ≥ 0.80), n (%)	72 (83.7%)	1 (100.0%)	15 (83.3%)	41 (87.2%)	15 (75.0%)	3 (100.0%)	9 (90.0%)	13 (76.5%)	12 (80.0%)	35 (85.4%)

IQR, interquartile range; NA, not applicable; PDC, proportion of days covered; SD, standard deviation; SMA, spinal muscular atrophy

^a^Age was measured at the index date

^b^PDC was calculated as the total number of days during which a patient had risdiplam available based on the reported days of supply of risdiplam beginning from the date of the first dispensing of risdiplam until their latest dispensing (plus days of supply), divided by the length of the adherence assessment period (i.e., study period) in days

#### Persistence and discontinuation

**Fig. 2:**
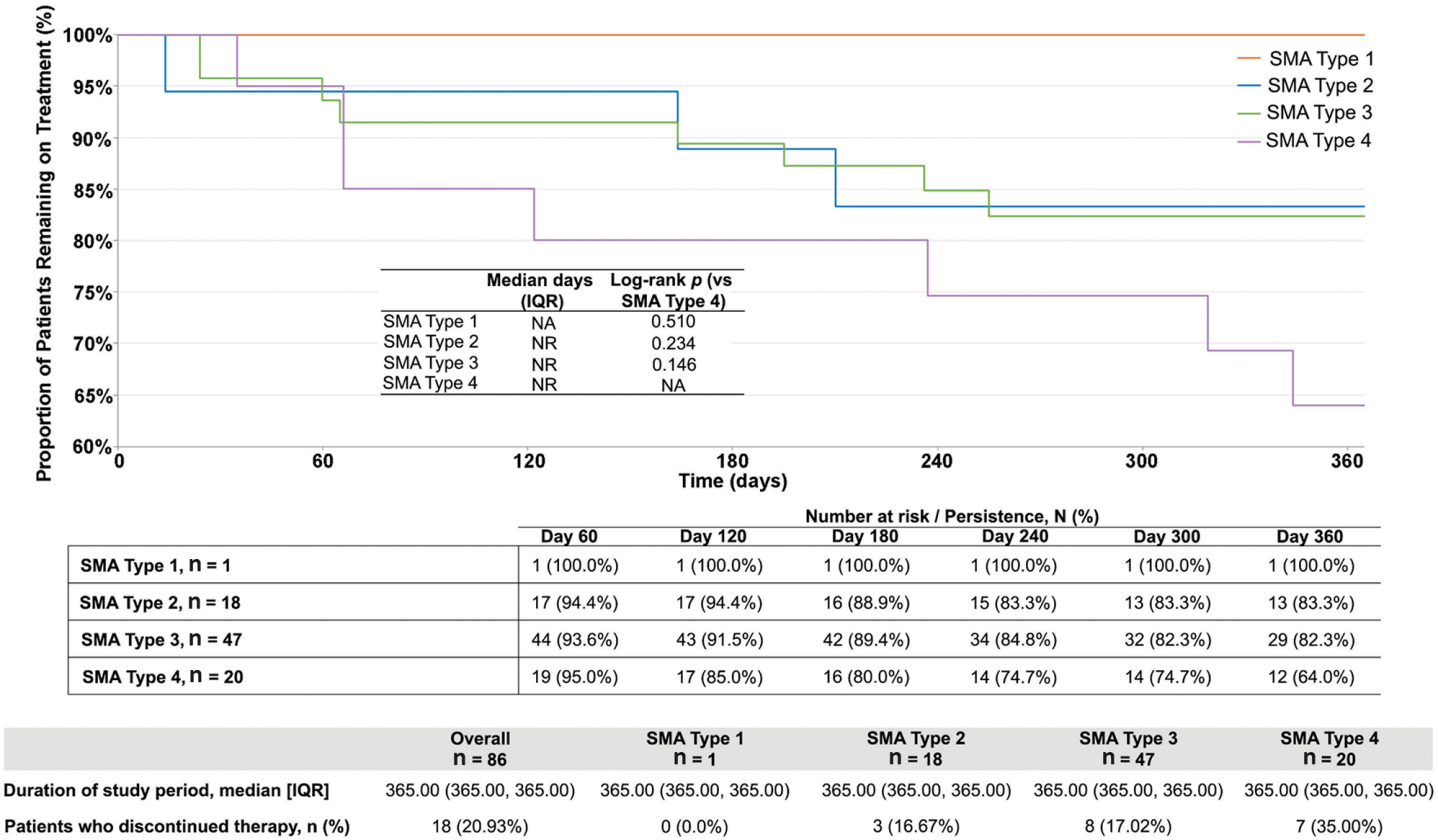
Kaplan Meier analysis of treatment persistence to risdiplam.^a, b^ IQR, interquartile range; NA, not applicable; NR, not reached; SD, standard deviation; SMA, spinal muscular atrophy. ^a^Risdiplam discontinuation was defined as no claim for risdiplam within 30 days after the previous days of supply was exhausted or switching to nusinersen and/or onasemnogene abeparvovec. ^b^Persistence was defined as the time from the index date (i.e., initiation of risdiplam) to the date of risdiplam discontinuation or end of study period

The rate of risdiplam persistence was similar for patients with SMA types 2 and 3 but declined rapidly for those with type 4 beginning 60 days after treatment initiation (Fig. 2). Within 1 year of risdiplam initiation, 18 patients (21%) discontinued risdiplam: three (17%) with SMA type 2, eight (17%) with SMA type 3, and seven (35%) with SMA type 4.

### Health care costs by adherence status

**Fig. 3:**
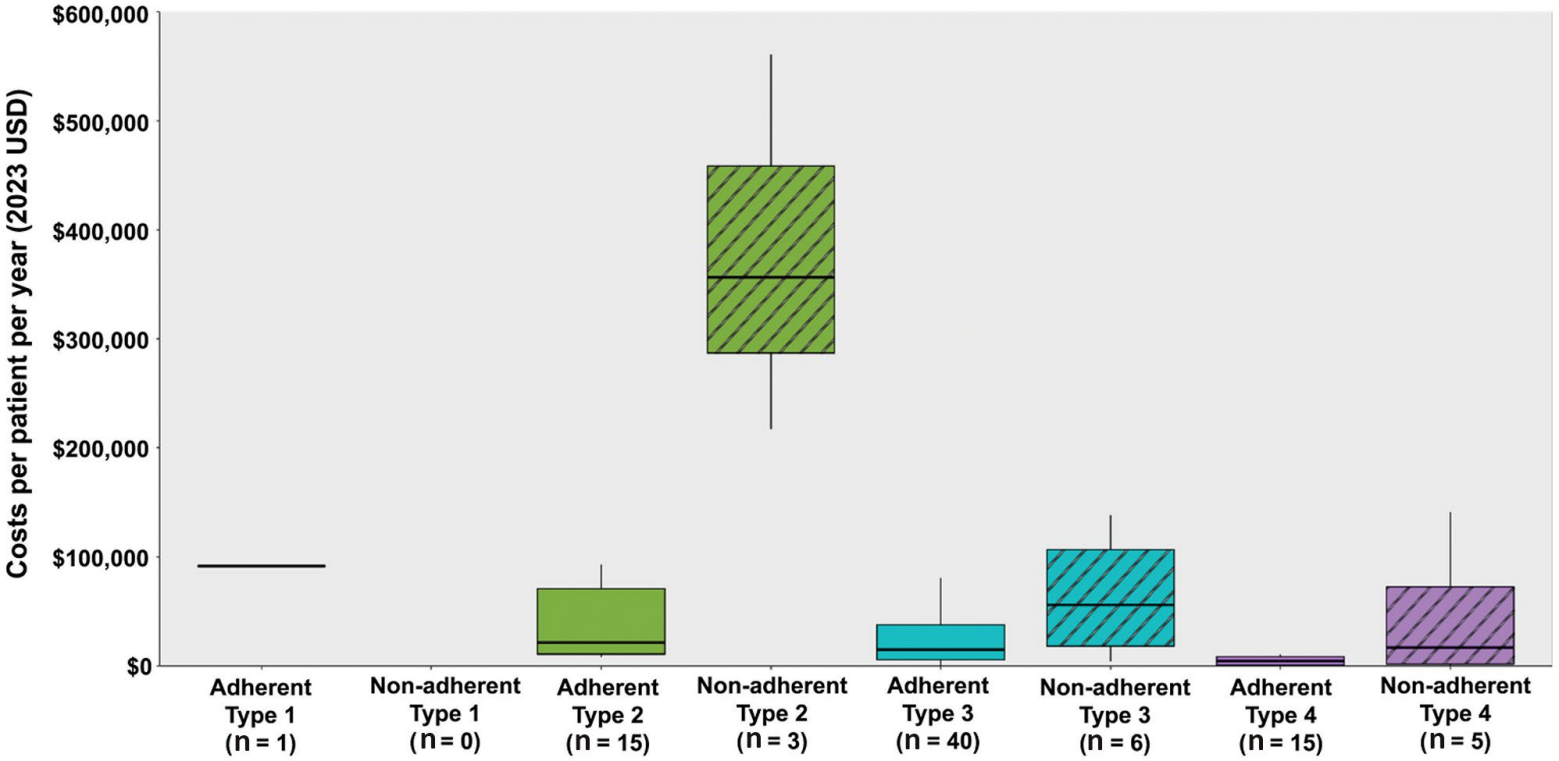
Total health care costs among patients with SMA who were adherent vs. not adherent to risdiplam.^a, b^*Note*: The figure displays the minimum, first quartile, median, third quartile, and maximum. The thick black line represents the median cost. The length of the box represents the 25th and 75th percentiles for costs (i.e., the interquartile range). The minimum and maximum health care cost, excluding outliers, is shown at the end of the whisker and at the start of the whisker, respectively. PDC, proportion of days covered; SMA, spinal muscular atrophy; USD, United States dollar. ^a^Adherence was defined as PDC ≥ 0.80. ^b^One outlier patient with excessive health care costs (>$4 million) was excluded from the analysis. This patient underwent multiple procedures that were unrelated to SMA within a relatively short timeframe

Median and mean all-cause total health care costs (PPPY) and all-cause medical service costs (PPPY) were greater for patients who were nonadherent to risdiplam than those who were adherent, a trend that extended across SMA types 2–4 (Table 3; Fig. 3). Specifically, median all-cause total health care costs PPPY of nonadherent versus adherent patients were 16-fold greater for SMA type 2 ($356,524.60 vs. $21,475.40), four-fold greater for SMA type 3 ($56,113.00 vs. $14,909.10), and three-fold greater for SMA type 4 ($16,817.00 vs. $4,594.10). Similarly, median all-cause medical service costs PPPY of nonadherent versus adherent patients were 11-fold greater for SMA type 2 ($354,854.50 vs. $21,402.80), five-fold greater for SMA type 3 ($54,218.80 vs. $10,188.70), and three-fold greater for SMA type 4 ($4,206.90 vs. $1,328.50).

**Table 3:** All-cause health care costs among patients with SMA who were adherent vs. not adherent to risdiplam

All-cause health care costs per patient per year (2023 USD), mean ± SD [median]^2^	SMA type 1	SMA type 2		SMA type 3		SMA type 4	
	Adherent^a^ (*n* = 1)	Adherent^a^ (*n* = 15)	Non-adherent (*n* = 3)	Adherent^a^ (*n* = 40)	Non-adherent (*n* = 6)	Adherent^a^ (*n* = 15)	Nonadherent (*n* = 5)
Total all-cause health care costs^b^	91,754.4 ± NA	49,061.8 ± 61,414.4 [21,475.4]	378,126.1 ± 172,674 [356,524.6]	34,249.2 ± 67,075.8 [14,909.1]	64,075.6 ± 55,904.2 [56,113]	15,583.4 ± 31,353.9 [4,594.1]	46,498.7 ± 60,638.7 [16,817]
Medical service costs^b^	91,204.6 ± NA	45,882.9 ± 60,925.1 [21,402.8]	376,187.5 ± 170,627.2 [354,854.5]	30,514.8 ± 66,468.8 [10,188.7]	62,668.9 ± 54,824 [54,218.8]	11,221.4 ± 29,712.2 [1,328.5]	32,651.7 ± 61,018.6 [4,206.9]
Mechanical ventilation support	12,389.7 ± NA	10,637.1 ± 24,242.5 [0]	159,882.9 ± 266,441.2 [12,186.4]	2,209.1 ± 7,594.2 [0]	27,106.3 ± 53,260.2 [0]	0 ± 0 [0]	0 ± 0 [0]
Nutritional support	34,984.1 ± NA	11,850.8 ± 31,034.4 [116.6]	162,689.6 ± 281,294.1 [568.8]	1,414.3 ± 3,614.7 [0]	8,372.6 ± 19,541 [0]	58.2 ± 201 [0]	0 ± 0 [0]
Pharmacy costs^c^	549.8 ± NA	3,178.9 ± 7,039.4 [1,132.7]	1,938.7 ± 2,046.9 [1,670.1]	3,734.4 ± 8,649.5 [535.3]	1,406.7 ± 1,539.2 [864.6]	4,362.1 ± 13,761.8 [195.7]	13,846.9 ± 30,460.1 [206.2]

IQR, interquartile range; NA, not applicable; PDC, proportion of days covered; SD, standard deviation; SMA, spinal muscular atrophy; USD, United States dollar

^a^Adherence was defined as PDC ≥ 0.80

^b^One outlier patient with excessive health care costs (>$4 million) was excluded from the analysis. This patient underwent multiple procedures that were unrelated to SMA within a relatively short timeframe

^c^Treatment costs of nusinersen and onasemnogene abeparvovec recorded in the medical claims and treatment costs of risdiplam recorded in pharmacy claims were excluded

**Fig. 4:**
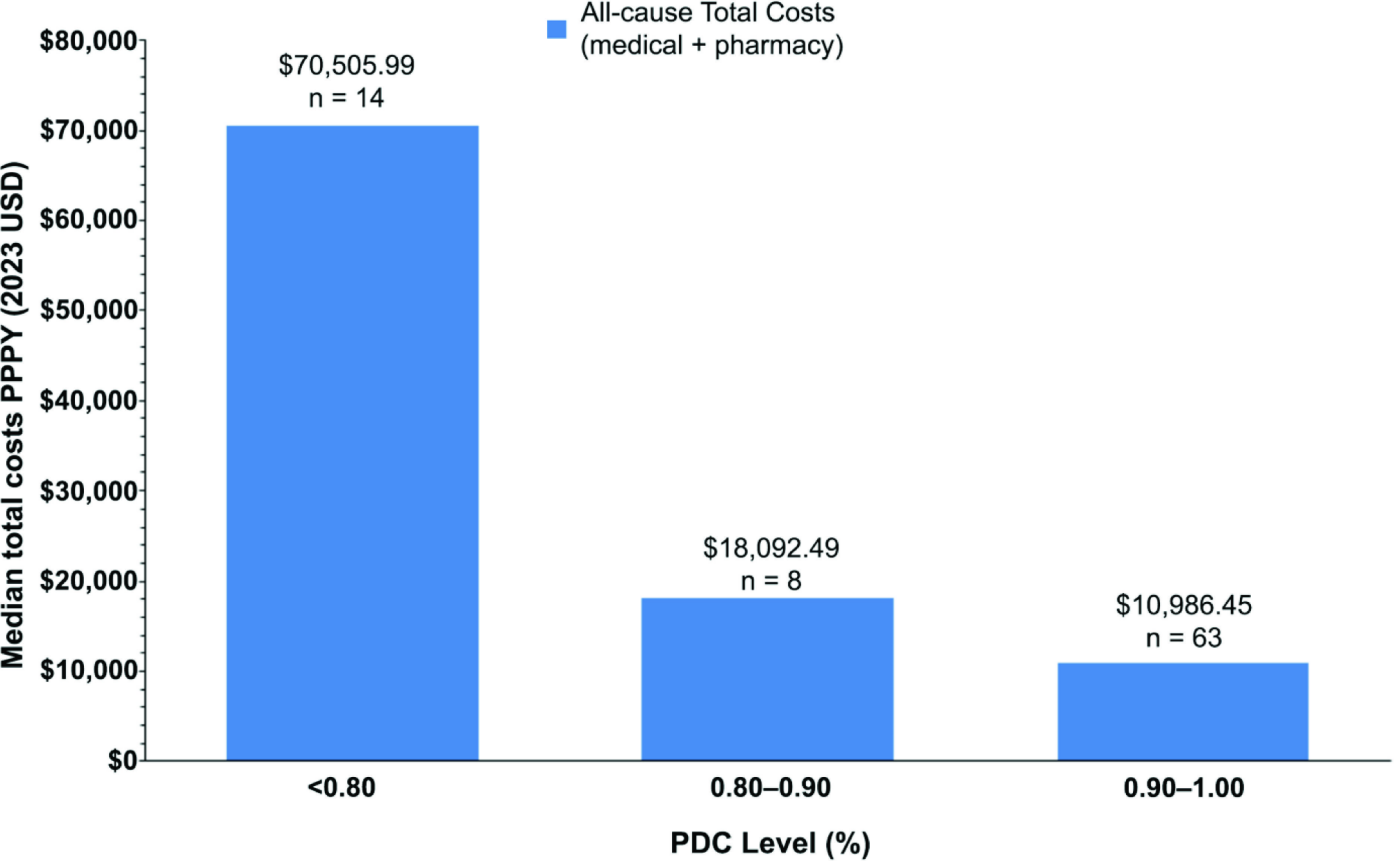
Total health care costs among SMA patients by level of PDC.^a, b^ PDC, proportion of days covered; PPPY, per-patient per-year; SMA, spinal muscular atrophy; USD, United States dollar. ^a^One outlier patient with excessive health care costs (>$4 million) was excluded from the analysis. This patient underwent multiple procedures that were unrelated to SMA within a relatively short timeframe. ^b^PDC level 0.80–0.90 means patients had a PDC level of ≥ 0.80 and < 0.90

Risdiplam-adherent patients with a PDC between 0.80 and 0.90 had approximately 1.6 times greater median all-cause total health care costs compared with patients with a PDC between 0.90 and 1.00 (PPPY: $18,092.50 vs. $10,986.50) (Fig. 4).

## Discussion

SMA is a complex disease that requires a multifaceted treatment plan, including medications, physical therapy, respiratory support, and various other interventions [25, 26]. Adherence to the prescribed treatment regimen is important for improving and maintaining motor function, and can substantially impact the treatment outcomes, health-related quality of life, and economic burden of patients with SMA [16]. The recent approval of risdiplam as a daily oral medication for patients with SMA prompts the need for evidence of its associated treatment patterns in the real world. This US claims database analysis bridged that gap by assessing the real-world adherence and persistence of patients with SMA treated with risdiplam, as well as the impact of nonadherence on their health care costs.

The results indicated that more than 80% of patients with SMA receiving risdiplam were adherent to the dosing regimen, which was consistent with findings from a prior study using a national specialty pharmacy database [27]. The level of adherence to the risdiplam dosing regimen was greater compared with nusinersen, as previous studies demonstrated mean adherence rates for nusinersen ranging from 71.8 to 75.6% across SMA types 1–3 and the adherence rate dropping to 41% at 56 weeks post-treatment initiation [16, 17]. The more invasive and distressing mode of administration for nusinersen may have compromised its adherence rate.

Despite the overall high adherence to the risdiplam dosing regimen, some variation across different age groups and SMA types were observed. For example, adherence to risdiplam was observed to be more than 80% in the age groups of 0–2 years (100%), 3–5 years (90%), 13–17 years (80%), and ≥ 18 years (84.5%), but under 80% in the age group of 6–12 years (76.5%). In addition, the adherence rate varied for patients with different SMA types, with the lowest adherence for patients with SMA type 4 (75%) and the highest for SMA type 1 (100%, albeit there is only one patient in the type 1 category). Nonadherence to risdiplam may be attributed to various factors, including those related to medications (e.g., polypharmacy, adverse events, costs); potential insufficient communication between the patient/caregiver and health care provider; insurance coverage; socioeconomic factors (e.g., financial hardship); or medically needed (e.g., disease progression requiring mechanical ventilation or nutritional support) and hence discontinuing risdiplam and switching to other treatments [28]. The variation in adherence rates across age groups could be attributed to the extent of involvement of parents or adult caregivers in administering the medication, as well as potential disease progression. Caregivers of the younger age group (e.g., 0–5 years old) are often acutely aware of the potential consequences of nonadherence, such as deteriorating health conditions or hospitalization, which may contribute to greater adherence rates for this age group. Likewise, adults tend to have a heightened awareness of the consequences of nonadherence, potentially contributing to greater adherence rates in this age group (≥ 18 years). We speculate that patients between 6 and 17 years of age may have a growing self-awareness, want more autonomy, and may not always follow the daily dosing routine [29, 30]. In addition, due to progressive muscle weakness, some patients with SMA type 2 may require mechanical ventilation as they age, which could lead to discontinuation of risdiplam and a switch to other treatments, consequently with a low adherence to the treatment.

SMA is a chronic, costly disease and nonadherence may further increase the economic impact to both patients and the US health care system. Because of the need for ongoing medical management and frequent hospitalizations, patients with SMA unsurprisingly incur substantially greater health care costs than those without the disease [31]. For example, a retrospective study using data from the US Department of Defense Military Healthcare System (2003–2012) estimated that the median total expenditure for patients with SMA over 7 years was approximately $80,000 greater than those for patients without SMA, with annualized costs of approximately $48,000 versus $1800, respectively [32]. Similarly, a retrospective study using data from the Truven Health Analytics MarketScan claims database (2012–2016) found that the mean annualized total net payment for inpatient admission was $118,609 for infants with SMA compared to $58.79 for infants without the disease, and a similar trend was observed for outpatient payments ($55,538 vs. $2,047, respectively) [33].

An important finding of this study is that patients with SMA types 2, 3, or 4 who were nonadherent to their risdiplam regimen incurred greater total health care costs compared with their adherent counterparts. These results were consistent with the findings of a recent (2021) study that reported that patients with SMA who were nonadherent to nusinersen incurred greater health care costs compared with adherent patients across SMA types 1–3 [16]. Together, these findings support the substantial economic impact related to nonadherence for patients with SMA who are treated with DMTs. In addition, our study found that patients who were highly adherent to risdiplam (i.e., those with a PDC of 0.90 to 1.00) incurred $7,106 lower median health care costs than patients who were adherent to a lesser extent (PDC between 0.80 and 0.90), highlighting the relationship between medical service costs and the extent of adherence to the treatment regimen.

Multiple factors can contribute to elevated health care expenses for nonadherent patients, a primary one being that medication nonadherence can negatively impact treatment effectiveness [34]. Worsened clinical outcomes may result in increased morbidity, lower health-related quality of life, and more frequent health care resource use (e.g., increased inpatient, outpatient, ED visits) resulting in excess health care costs [35]. Nonadherent patients across SMA types 2−4 incurred higher costs for medical services compared with adherent patients. The cost differences dramatically increased with disease severity and were substantially greater for nonadherent patients with SMA type 2 (total medical service costs PPPY: $376,188), largely attributable to the need for mechanical ventilation and nutritional support. This finding underscores the critical role of treatment adherence in mitigating potentially costly complications and providing disease control, thereby reducing morbidity as well as health care resource utilization and costs.

Finally, it is worth noting that although the traditional definition of adherence as 80% PDC is generally acceptable for many chronic diseases (i.e., hypertension [36], diabetes [37]), this may not be the case for potentially rare, progressive disorders like SMA in which even minor treatment gaps may substantially impact disease management. Unfortunately, medication adherence rates for individuals with rare diseases are among the lowest for any diseases, and the rates were reported to be 58–65% in a global study of nonadherence conducted in the United States, United Kingdom, Germany, Australia, and New Zealand [38]. Few studies have examined the impact of nonadherence for individuals with rare diseases, although rare diseases have disproportionate mortality rates. For example, a population-based registry study in Italy found that although only 5% of people have been diagnosed with a rare disease, they accounted for 13% of deaths annually [39]. Poor or even conventionally ‘good’ adherence may lead to irreversible functional decline or potentially life-threatening complications for patients with SMA. More assessments are needed to better understand the optimal adherence level for patients with SMA.

The results of this study should be interpreted in the light of several limitations, some of which are common for retrospective claims database analyses. First, this study used a sample of commercially insured individuals; thus, the results may have limited generalizability to Medicaid and/or uninsured populations. Second, we defined exposure to risdiplam using the days of supply records in the pharmacy claims, which may not accurately reflect whether the medication was actually taken by the patient. Third, as administrative claims data are collected for payment rather than research purposes, there may be billing inaccuracies and missing data related to miscoding of medical diagnoses. Thus, the analysis is vulnerable to coding inaccuracies. Fourth, there is a potential risk of misclassification of SMA types based on an established algorithm as such information is not available in health insurance claims data. In addition, SMA is a rare disease, which limited the sample size of eligible patients for this study. Only one patient with SMA type 1 receiving risdiplam was captured. One possible reason is that risdiplam was first approved for adults and children 2 months and older on August 7, 2020, and later for children under 2 months of age (May 31, 2022), which was roughly 1 month before the end of the available data period, limiting this patient population for study inclusion. In conclusion, the analyses conducted in this study were descriptive in nature and therefore causal relationships could not be ascertained. Future studies with a larger sample size and longer follow-up period may allow for a statistical comparison of health care costs between adherent and nonadherent patients while adjusting for age and other confounding factors.

## Conclusions

This study provides a real-world assessment of treatment adherence and discontinuation, along with all-cause health care costs by adherence status, for patients with SMA who were treated with risdiplam. Nonadherence to risdiplam was observed in the first year of treatment, especially for patients with SMA type 4 and patients aged 6 to 12 years. Adherence to risdiplam was associated with lower all-cause health care costs. This difference was more pronounced for patients with SMA type 2, underscoring the importance of better treatment adherence, which could result in lower health care resource utilization and associated costs, particularly for younger children. For adherent patients, patients with PDC between 0.90 and 1.00 had lower median health care costs compared with patients with PDC of 0.80 to 0.90. Additional research with a larger sample size and longer follow-up are needed to further examine the long-term impact of real-world adherence to risdiplam on patient outcomes and to identify effective interventions for improving adherence.

## Electronic supplementary material

Below is the link to the electronic supplementary material.

Supplementary Material 1

## Data Availability

This information is an estimate derived from the use of information under license from the following IQVIA information service: PharMetrics^®^ Plus for the period December 10, 2021 – December 9, 2024. IQVIA expressly reserves all rights, including rights of copying, distribution, and republication. Data may be available upon request and may be subject to a data sharing agreement.

## Abbreviations

DME: Durable medical equipment
DMT: Disease-modifying treatment
ED: Emergency department
FDA: Food and Drug Administration
ICD-10-CM: International Classification of Disease, Tenth Revision, Clinical Modification
IQR: Interquartile range
NR: Not reached
PDC: Proportion of days covered
PPPY: Per-patient per-year
SD: Standard deviation
SMA: Spinal muscle atrophy
*SMN1*: *Survival motor neuron 1* gene
US: United States
USD: United States dollars
