# Supplementary material for: Risdiplam utilization, adherence, and associated health care costs for patients with spinal muscular atrophy: a United States retrospective claims database analysis
Source: Orphanet J Rare Dis. 2024 Dec 30;19:494. doi: 10.1186/s13023-024-03399-0 (PMC11684252; doi:10.1186/s13023-024-03399-0)
Supplement: Supplementary file 1 — Supplementary Material 1 [file 13023_2024_3399_MOESM1_ESM.docx]

# SUPPLEMENTAL MATERIAL

## Figure S1. Study design


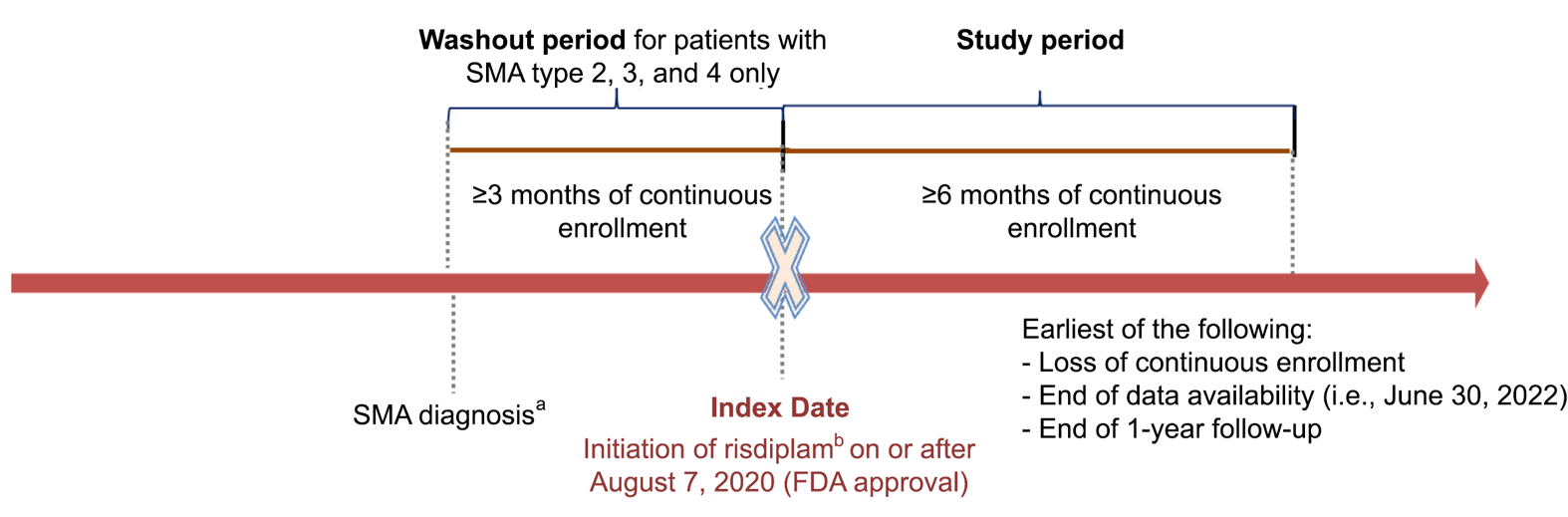


Note: The figure is for illustrative purposes only (not scaled).

FDA, Food and Drug Administration; SMA, spinal muscle atrophy.

^a^SMA diagnosis was required anytime between January 1, 2020, and June 30, 2022.

^b^The initiation of risdiplam was identified based on the first claim for treatment with risdiplam.

## Table S1. Algorithm for the identification of SMA types

| **Criterion** | **Age at first SMA diagnosis** | **Age at current onset** | **Characteristic** | **SMA type** |
| --- | --- | --- | --- | --- |
| 1 | 0–1 year | 0–3 years | – | Type 1 |
| 2 | 2 years | 2–13 years | – | Type 2 |
| 3 | ≥3 years, <21 years | ≥3 years | – | Type 3 |
| 4 | ≥21 years | ≥21 years | Scoliosis | Type 3 |
| 5 | ≥21 years | ≥21 years | Gastrostomy | Non SMA |
| 6 | ≥21 years, <35 years | ≥21 years | No scoliosis or gastrostomy | Type 4 |
| 7 | ≥35 years | ≥35 years | – | Type 4 |
| **Reclassification of SMA types for patients with DME** | | | | |
| 8 |  | >50 years | Cane or walker at age >50 years | Type 4 |
| 9 |  |  | Walker (if #8 does not apply) | Type 3 |
| 10 |  |  | Pediatric wheelchair (tilt-in-space) (if #8–9 do not apply) | Type 2 |
| 11 |  | ≤17 years | Pediatric wheelchair at age <7 years (if #8–10 do not apply) | Type 2 |
| 12 |  | ≤17 years | Pediatric wheelchair at age ≥7 years (if #8–11 do not apply) | Type 3 |
| 13 |  | >60 years | Manual adult wheelchair at age >60 years (if #8–12 do not apply) | Type 4 |
| 14 |  |  | Manual adult wheelchair (if #8–13 do not apply) | Type 3 |
| 15 |  |  | Braces (if #8–14 do not apply) | Type 2 |

DME, durable medical equipment; SMA, spinal muscular atrophy.

## Table S2. Diagnosis and procedure codes used for the identification of SMA types

| **Diagnosis/procedure/DME** | **Code type** | **Codes** |
| --- | --- | --- |
| Scoliosis | ICD-10-CM | M40.29, M40.3x, M40.4x, M40.5x, M41.1x, M41.2x, M41.3x, M41.4x, M41.5x, M41.8x, M41.9x, M43.8x, M96.3, M96.4 |
| Gastrostomy | CPT | 43246, 43280, 43653, 43750, 43760, 43830, 43832, 43870, 49440, 49450, 49460, 49465 |
|  | ICD-10-CM | Z93.1x |
| Cane | HCPCS | E0100, E0105 |
| Walker | HCPCS | E0135, E0141, E0143, E0147, E0149 |
| Pediatric wheelchair (tilt-in-space) | HCPCS | E1231, E1232, E1233, E1234 |
| Pediatric wheelchair | HCPCS | E1235, E1236, E1237, E1238 |
| Manual adult wheelchair | HCPCS | E1161 |
| Braces | HCPCS | L1902, L1904, L1907, L1910, L1920, L1930, L1960, L1970, L4350, L4397 |

CPT, Common Procedure Terminology; DME, durable medical equipment; HCPCS, Healthcare Common Procedure Coding System; ICD-10-CM, International Classification of Diseases, Tenth Revision, Clinical Modification.

## Table S3. Codes to identify SMA related treatments

| **Treatment** | **Code type** | **Codes** | **Description** |
| --- | --- | --- | --- |
| Onasemnogene abeparvovec | NDC | 71894-0110-01  71894-0115-01  71894-0120-02  71894-0121-03  71894-0122-03  71894-0123-03  71894-0124-04  71894-0125-04  71894-0126-04  71894-0127-05  71894-0128-05  71894-0129-05  71894-0130-06  71894-0131-06  71894-0132-06  71894-0133-07  71894-0134-07  71894-0135-07  71894-0136-08  71894-0137-08  71894-0138-08  71894-0139-09  71894-0140-09  71894-0141-09 | Zolgensma (onasemnogene abeparvovec) |
|  | HCPCS | J3399 | Injection, onasemnogene abeparvovec-xioi, per treatment, up to 5x10^15 vector genomes |
| Nusinersen | NDC | 64406-058-01  71860-396-01 | Spinraza (nusinersen) |
|  | HCPCS | C9489  J2326 | Injection, nusinersen, 0.1 mg |
| Risdiplam | NDC | 50242-0175-05  50242-0175-07 | Evrysdi (risdiplam) |

HCPCS, Healthcare Common Procedure Coding System; NDC, National Drug Code.
